# Heightened Serum Mitochondrial Biomarkers; FGF21 and NOS in Pediatric Anemia and a Negative Correlation between GDF15 and Serum Ferritin

**DOI:** 10.3390/jcm13154403

**Published:** 2024-07-27

**Authors:** Hatice Mine Çakmak, Merve Alpay, Cansu Mahdızadeh, Seray Çevikel Özalp, Sevim Türay, Şükriye Özde, Kenan Kocabay

**Affiliations:** 1 Pediatric Hematology-Oncology, Duzce University School of Medicine, Konuralp Provinence, 81620 Duzce, Turkey; 2 Biochemistry, Duzce University School of Medicine, Konuralp Provinence, 81620 Duzce, Turkey; 3 Pediatrics, Duzce University School of Medicine, Konuralp Provinence, 81620 Duzce, Turkey; 4 Pediatric Neurology, Duzce University School of Medicine, Konuralp Provinence, 81620 Duzce, Turkey

**Keywords:** NOS, GDF15, FGF21, anemia, child

## Abstract

**Objective:** Mitochondrial dysfunction is closely linked to chronic disorders. This study aims to explore the correlation between pediatric anemia and mitochondrial markers, specifically fibroblast growth factor 21 (FGF21), growth/differentiation factor 15 (GDF-15), and nitric oxide synthase (eNOS). **Method:** This study included 66 children, with 34 diagnosed with anemia and 32 in the healthy control group. Statistically significant biomarkers were determined through cutoff levels. **Results:** Among the participants, 34 children were classified as anemic, while 32 were categorized as healthy. The study revealed that FGF21 levels ≥ 0.745 pg/mL and eNOS levels ≥ 1.265 µg/mL predicted anemia. Hemoglobin levels exhibited a negative correlation with FGF21 (r = −0.381; *p* = 0.002) and eNOS levels (r = −0.462; *p* < 0.001). Furthermore, a significant negative correlation was observed between GDF-15 and ferritin (r = −0.311; *p* = 0.019), while eNOS levels correlated positively with folate (r = 0.313; *p* = 0.019). **Conclusions:** Anemia induced elevated mitochondrial biomarkers; FGF21 and eNOS levels. The findings suggest that the long-term ramifications of anemia in childhood may be associated with mitochondrial dysfunction.

## 1. Introduction

Mitochondria, the cellular organelles responsible for energy production, may experience dysfunction resulting in the disruption of normal functions such as apoptosis and pyrimidine biosynthesis [1]. Some inherited mitochondrial diseases are Barth Syndrome, Person syndrome, and MLASA (mitochondrial myopathy, lactic acidosis, and sideroblastic anemia). Additionally, mitochondrial dysfunction was reported to be involved in acquired disorders such as diabetes, cancer, cardiovascular disorders, Alzheimer’s disease, exercise intolerance, and anemia [2,3]. Anemia leads to mitochondrial dysfunction by reducing the NO pool and increasing free oxygen radicals. Vascular and endothelial NOS upregulation is essential for compensating the NO pool [4]. Endothelial nitric oxide synthetase (eNOS) is found in the red blood cell membrane. NO (nitric oxide) has critical roles in vascular tonus, insulin secretion, respiratory tonus, angiogenesis, and neuronal development. Blood loss and hemolytic anemia are linked to dysregulation of redox homeostasis, NO- scavenging, and coronary vascular disease [5]. In moderate blood loss, the circulating NO pool is decreased, and erythroid dysfunction occurs. RBC decrease and dysfunction lead to reduced NO bioavailability, which alters coronary vessels [6]. Depletion in iron stores leads to mitochondrial dysfunction associated with increased lactate, lactate/pyruvate, and low L-carnitine levels [7]. Iron depletion in human erythroid progenitors alters mitochondrial function; intermediate iron restriction causes mitochondrial dysfunction by inhibiting mitochondrial aconitase and increases mitochondrial reactive oxygen species (superoxide/hydrogen peroxide) in erythroid progenitors. When NO reacts with O_2_, peroxynitrite is produced, which damages mitochondria. Deficiencies in cofactors of mitochondrial enzymes such as iron, zinc, copper, and riboflavin in heme biosynthesis can cause mitochondrial decay [2,7].

FGF21 and GDF15 are well-known mitochondrial biomarkers that showed remarkable elevations in mitochondrial diseases, respectively [8,9,10]. Fibroblast Growth Factor (FGF) is a protein family that plays a significant role in various biological processes such as cell growth, proliferation, differentiation, and tissue repair. FGF21 plays a vital role in processes such as energy and lipid metabolism in the body and can contribute to correcting mitochondrial dysfunction [10,11]. GDF15 (Growth/differentiation factor 15) was first identified as Macrophage inhibitor cytokine-1 or MIC-1. Under normal conditions, GDF-15 is released in low concentrations in most tissues. The function of GDF-15 is regulating inflammatory pathways and apoptosis, cell repair, and cell growth, which are biological processes observed in cardiovascular and neoplastic disorders [12]. The change in serum FGF21 and GDF-15 in anemia or iron deficiency anemia is not reported.

A significant gap exists in the current literature regarding the association between mitochondrial dysfunction and anemia in a dominant pediatric population with iron deficiency anemia, as evidenced by the absence of studies utilizing the markers of FGF21, GDF-15, and NOS. This study seeks to address this gap by examining the demographic profiles, laboratory parameters, and serum levels of FGF21, GDF15, and NOS in both anemic and non-anemic pediatric subjects. The study aims to determine the discriminatory thresholds, specificity, and sensitivity of the novel biomarkers FGF21, GDF15, and NOS in diagnosing mitochondrial dysfunction associated with pediatric anemia.

## 2. Materials and Methods

In November–December 2022, the Pediatric Hematology-Oncology outpatient clinic at our University, Research, and Training Hospital admitted 66 children aged 6 months to 17 years. These patients underwent preliminary screening of their hemoglobin levels. Children whose hemoglobin levels were below two standard deviations for their respective ages were classified into the anemia group (*n* = 34), while the others were included in the non-anemic group (*n* = 32). Exclusion criteria consisted of severe chronic ailments such as heart failure, chronic renal disease necessitating dialysis, respiratory failure, metabolic disorders, cytopenia, or pancytopenia.

Furthermore, the study group included an evaluation of the children’s age, anemia duration (months), hemoglobin (g/dL), red blood cell count (×10^6^/mm^3^), average cell volume (fL), folate (ng/mL), and vitamin B12 (pg/mL), along with serum iron levels (μg/dL), iron binding capacity (mcg/dL), and ferritin (ng/mL) for all individuals. Blood samples were collected following a fasting period of at least 12–14 h, adhering to strict sterilization protocols. The levels of FGF21 (pg/mL), GDF15 (pg/mL), and NOS (µg/mL) were determined using the IDS (Immunology Diagnostic Systems, Tyne and Wear, UK) analyzer B0728 autoanalyzer device, employing the enzymatic colorimetric method. The blood samples were centrifuged at the biochemistry laboratory at 4 °C and 3000 rpm for 15 min under aseptic conditions and stored at −80 °C until the study period. Colorimetric ELISA commercial kits were utilized for analyzing and implementing study protocols for each FGF21, GDF15, and NOS parameter.

The sample size was determined using G*Power V. 3.1.9.6. GDF15, FGF21, and NOS values were calculated at a 95% confidence level (1 − α) and 95% test power (1 − β) with d = 0. Based on a one-way hypothesis, an effect size of 0.341 was calculated, and the total number of cases in the study was 26, with 13 in each group. Subsequently, when ten individuals were included in each group, the test yielded a result of 89–23%. Data analysis was carried out using IBM SPSS V23. Normal distribution conformity was assessed through the Shapiro–Wilk Test and Kolmogorov–Smirnov Test. Categorical variable comparisons were conducted using the Pearson Chi-Square Test with Yates Correction and Fisher’s Exact Test. Additionally, the Independent Samples *t*-test was utilized to compare normally distributed variables between groups, while the Mann–Whitney U Test was employed for non-normally distributed variables. Furthermore, Pearson’s correlation coefficient was used to examine the relationship between normally distributed variables, while Spearman’s correlation coefficient was used for non-normally distributed variables. Lastly, a Roc analysis was performed to determine the cutoff point for parameters predicting anemia. The analysis results were presented, detailing frequency (percentage) for categorical variables, average ± standard deviation, and median with a range of values for quantitative variables. Statistical significance was set at *p* < 0.005. The correlation of FGF21, GDF15, and NOS parameters with the serum ferritin, folate, vitamin B12, and iron was calculated. The research ethics were approved by the University Association ethics committee on 7 November 2022, with decision number 2022/174. Informed consent forms were signed by all participating patients.

## 3. Results

In the study cohort comprising 66 children, 34 were classified under the anemia group, while 32 were categorized under the non-anemic group. Analysis of demographic characteristics revealed a significantly higher proportion of females in the anemia group compared to the non-anemic group (82.4% vs. 43.8%, respectively; *p* = 0.003). Moreover, a history of anemia was more prevalent in the anemia group compared to the non-anemic group (23.5% vs. 0%, respectively; *p* = 0.005). However, there was no significant difference in the distribution of concurrent diseases between the two groups (*p* = 0.057). Iron deficiency emerged as the primary cause of anemia in the study cohort, accounting for 66.7% *(n =* 14) of cases. Additionally, other contributing factors including inflammation, chronic disease, and non-immune hemolytic anemia were identified (see Table 1).

The mean ages of the participants, as well as their serum folate, Vitamin B12, iron-binding capacity, and ferritin levels, did not show statistically significant differences between the anemia and non-anemia groups (see Table 2). However, the anemia group exhibited a significant decrease in hemoglobin, red blood cell count, mean cell volume, and iron levels (see Table 2). In comparing FGF21, GDF15, and NOS levels, it was found that the mean levels of FGF21 (1.24 ± 0.53 pg/mL vs. 0.95 ± 0.66 pg/mL, respectively) (*p* = 0.008) and NOS (1.72 ± 0.59 µg/mL vs. 1.43 ± 0.49 µg/mL, respectively) (*p* = 0.036) were significantly higher in the anemia group, whereas GDF15 levels showed similar results (see Table 2).

Hemoglobin levels correlated negatively with those of FGF21 (r = −0.381; *p* = 0.002) and NOS levels (r = −0.462; *p* < 0.001). A significant negative correlation was found between GDF15 and ferritin (r = −0.311; *p* = 0.019). NOS correlated positively with folate (r = 0.313; *p* = 0.019). No correlation was found between these biomarkers and vitamin B12 or iron (*p* > 0.050) (Table 3).

**Figure 1:**
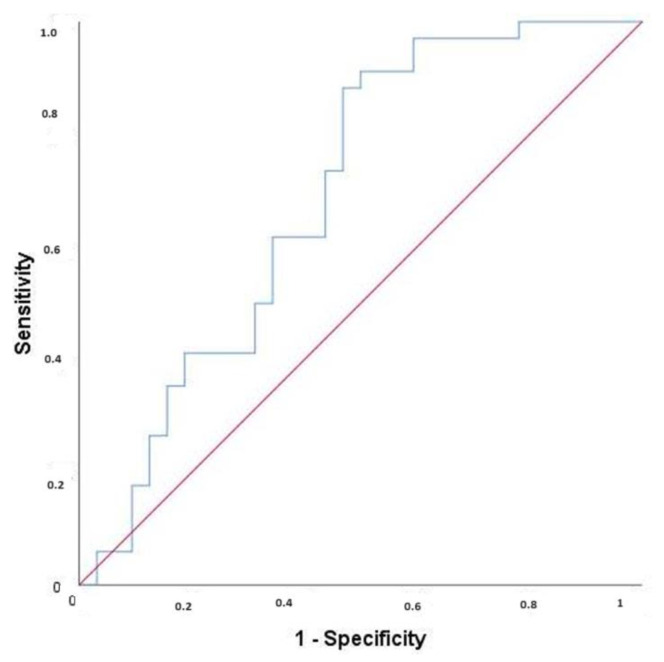
ROC curve for FGF21.

**Figure 2:**
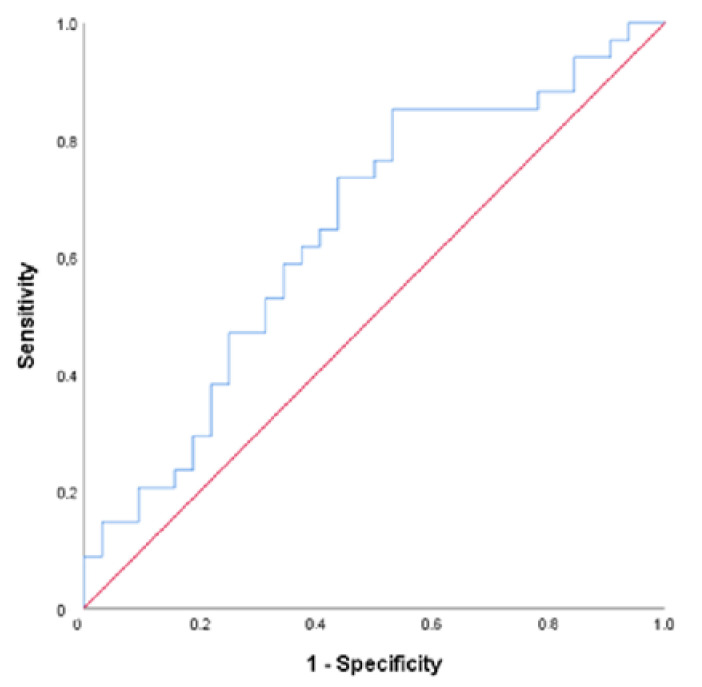
ROC curve for NOS.

A significant cut-off point of ≥0.745 pg/mL for FGF21 was found to predict anemia (AUC = 0.690; *p* = 0.008) with a sensitivity of 88.24% and specificity of 53.12%. PPV was 66.67%, and NPV was 80.95% of this value for the prediction of anemia (Figure 1). We could find no cut-off point for GDF15 (AUC = 0.494; *p* = 0.928). A cut-off value of ≥1.265 (µg/mL) was statistically significant for NOS with a sensitivity of 73.53%, specificity of 56.25, PPV of 64.1%, and NPV of 66.67% (AUC = 0.647; *p* = 0.040) (Figure 2).

It is important to note that no correlation was found between these biomarkers and vitamin B12 or iron (*p* > 0.050), indicating a potential area for further investigation.

The statistical significance of our findings provides reassurance about the validity of the research. A cut-off point of ≥0.745 pg/mL for FGF21 was found to predict anemia (AUC = 0.690; *p* = 0.008) with a sensitivity of 88.24% and specificity of 53.12%. The PPV was 66.67%, and the NPV was 80.95% of this value for the prediction of anemia (Figure 1). We could find no cut-off point for GDF15 (AUC = 0.494; *p* = 0.928). A cut-off value of ≥1.265 (µg/mL) was statistically significant for NOS with a sensitivity of 73.53%, specificity of 56.25, PPV of 64.1%, and NPV of 66.67% (AUC = 0.647; *p* = 0.040) (Figure 2).

In our analysis, we observed elevated levels of FGF21 (≥0.745 pg/mL) and NOS (≥1.265 µg/mL) in children primarily diagnosed with iron deficiency anemia as compared to non-anemic children. Furthermore, we discovered a notable negative correlation between GDF15 and ferritin (r = −0.311; *p* = 0.019), while NOS exhibited a positive correlation with folate (r = 0.313; *p* = 0.019) (Table 4).

In our study, for children mostly with iron deficiency anemia, we found that FGF21 (with a cut-off level of ≥0.745 pg/mL) and NOS (with a cut-off grade of ≥1.265 µg/mL) levels increased compared to non-anemic children. However, a significant negative correlation was found between GDF15 and ferritin (r = −0.311; *p* = 0.019). Conversely, NOS correlated positively with folate (r = 0.313; *p* = 0.019). Child onset anemia is an area of investigation concerned with the association of serum eNOS and FGF21 elevation.

## 4. Discussion

It has been confirmed that translational defects in mitochondrial and nuclear DNA, typically seen in mitochondrial diseases involving skeletal muscle, lead to increased FGF21/GDF15 levels. FGF21, a mitochondrial stress biomarker, is also elevated in obesity, type 2 diabetes, coronary heart disease, and chronic kidney disease [13]. Our research demonstrates that anemia is linked to mitochondrial dysfunction, as indicated by elevated levels of FGF21 and eNOS. Our study is the first to show the increase of FGF21 in pediatric patients with anemia, supporting the presence of mitochondrial dysfunction in an anemic pediatric population where iron deficiency anemia is the majority. Our ROC curve analysis revealed that FGF21 is more effective than eNOS in distinguishing between anemic and non-anemic individuals.

Additionally, we found that low ferritin levels were negatively associated with increased GDF15, which is a biomarker originating from erythroid progenitors, denoted ineffective erythropoiesis, and participated in the regulation of iron metabolism by suppressing hepcidin [8]. Despite its various associations with erythroid disorders, the precise function of GDF15 in erythropoiesis remains subject to debate. Our study, in contrast with prior research, demonstrated an inverse correlation between serum GDF15 and ferritin levels, with comparable findings observed in both anemic and non-anemic children. Notably, low ferritin levels (<12 ng/mL) were linked to abnormal mitochondrial function, as verified through mitochondrial function tests utilizing Lactate, L-Carnitine, and serum lactate/serum pyruvate ratio assessments. Further investigation revealed that anemia associated with chronic diseases and iron deficiency anemia independently induced a marked increase in GDF15. Conversely, isolated iron deficiency anemia did not significantly alter GDF15 levels. In patients aged over 60, GDF15 emerged as a standalone predictor of anemia [14]. Within our study group, iron deficiency anemia was prevalent, with a predominance of females among anemia sufferers. Despite observations of similar levels of GDF15, Vitamin B12, total iron binding capacity, and ferritin in both anemic and non-anemic cohorts, notable reductions were noted in serum iron levels and mean corpuscular volume (MCV) among anemic children. Additionally, a noteworthy negative correlation between GDF15 and ferritin was identified, contradicting outcomes observed among the elderly population [15]. Furthermore, unlike the senior demographic, no apparent association between GDF15 and anemia which was observed in the pediatric group in our research, which may be due to the limited small number of patients.

We report that serum eNOS levels were elevated in pediatric patients with anemia. NOS levels correlated positively with folate and negatively correlated with hemoglobin levels. A cut-off value for NOS, ≥1.265 (µg/mL), was found for detecting anemia. However, NOS levels were not associated with serum ferritin and iron levels. An increment in nitric oxide plays a role in anemia. When NO levels increase, Hb decreases—hemolysis causes NO reduction and endothelial dysfunction. Jeffrey’s study demonstrated that increased free hemoglobin causes NO reduction [16]. In contrast to our study, Odemis et al. reported that iron-deficient children had higher nitrate and nitrite levels and suggested the correlation between nitric oxide and ferritin [17]. As a signal molecule of the endothelium, NO is produced by NOS. Eritrocytes control systemic NO bioavailability by creating ATP in hypoxia and sheer stress. In moderate blood loss, the circulating NO pool is decreased, and erythroid dysfunction occurs. RBC decrease and dysfunction lead to reduced NO bioavailability, which alters coronary vessels. Folate increases NO bioavailability by increasing NO coupling and providing bioavailable NOS [18]. Our results are consistent with previous studies in showing serum folate levels’ role in increasing serum eNOS levels.

The study findings suggest that pediatric patients diagnosed with anemia exhibit elevated serum eNOS levels. These levels demonstrate a positive correlation with folate and a negative correlation with hemoglobin levels. A determined cutoff value of eNOS, ≥1.265 (µg/mL), has been identified as effective in detecting anemia. Conversely, eNOS levels show no significant association with serum ferritin and iron levels. Of note is the role of nitric oxide (NO) in the context of anemia, where elevated NO levels correspond to diminished hemoglobin (Hb) levels. The reduction of NO due to hemolysis leads to endothelial dysfunction. Notably, Jeffrey’s research indicates that increased free hemoglobin contributes to NO reduction [17]. In contrast, research by Odemis et al. suggests that iron-deficient children exhibit higher nitrate and nitrite levels, implying a correlation between nitric oxide and ferritin [18]. As a signaling molecule of the endothelium, NO is produced by NOS. Erythrocytes modulate systemic NO bioavailability by generating ATP in the presence of hypoxia and sheer stress. In scenarios involving moderate blood loss, a decline in the circulating NO pool results in erythroid dysfunction. This decrease in red blood cells (RBCs) and decreased functioning of red blood cells leads to reduced NO bioavailability, impacting coronary vessels. Folate serves to enhance NO bioavailability by promoting NO coupling and providing bioavailable NOS [17,18]. These outcomes are in line with prior studies suggesting the involvement of serum folate levels in augmenting serum eNOS levels.

In mice models, iron deficiency did not cause impairment in the activity of the skeletal muscles’ mitochondrian respiratory chain complex I, or reduction in physical endurance [19].

The study’s limitations encompass its reliance on a small patient cohort and the inclusion of children with varied anemia etiologies: iron deficiency (66.7%), inflammation anemia (14.3%), non-immune hemolytic anemia (4.8%), and anemia of chronic disease (14.3%). Future research stands to benefit from larger patient cohorts including only iron deficiency and iron deficiency anemia to assess the enduring impacts of mitochondrial dysfunction due to anemia.

## 5. Conclusions

The findings of our research reveal the correlation between anemia-related elevation in FGF21 and NOS levels and the potential development of mitochondrial dysfunction. Reduced serum ferritin levels caused elevated GDF15 levels that possibly caused impairment in mitochondrial function. These results support mitochondrial impairment in humans. Further studies are required to investigate the mechanism and consequences of these changes in mitochondrial metabolism, and the effect of iron therapy on iron deficiency and iron deficiency anemia.

## Figures and Tables

**Table 1:** Demographics of anemic patients and the controls.

	Groups		Test Statistics	*p*
	Anemia n (%)	Non-Anemic n (%)		
Sex				
Male	6 (17.6)	18 (56.3)	9.013	0.003 *
Female	28 (82.4)	14 (43.8)		
Diagnosis of anemia				
Iron deficiency anemia	14 (66.7)	-	-	-
Inflammation anemia	3 (14.3)	-	-	
Non-immune hemolytic anemia	1 (4.8)	-	-	
Chronic disease anemia	3 (14.3)	-	-	
Unknown	13 (38.2)			
History of anemia				
Absent	26 (76.5)	31 (100)	-	0.005 **
Present	8 (23.5)	0 (0)	-	
Concurrent diseases				
Allergic rhinitis	1 (6.7)	0 (0)	21.867	0.057 ***
Growth failure	1 (6.7)	1 (11.1)		
Epilepsy	3 (20.1)	0 (0)		
Genetic diseases	0 (0)	1 (11.1)		
Hypothyroidism	1 (6.7)	0 (0)		
Hemangioma	1 (6.7)	0 (0)		
Hypertension	1 (6.7)	0 (0)		
Immune deficiency	0 (0)	1 (11.1)		
Idiopathic thrombocytopenic purpura	0 (0)	2 (22.2)		
Migraine	4 (26.7)	0 (0)		
Nephrolithiasis	1 (6.7)	0 (0)		
Obesity	0 (0)	4 (44.4)		
Thrombocytopenia	2 (13.3)	0 (0)		

* Yates Correction; ** Fisher’s Exact Test; *** Pearson Ki-Kare Test.

**Table 2:** Comparison of serum NOS, GDF15, FGF21, and other laboratory values between anemia and control groups.

	Groups				Test Statistics	*p*
	Anemia		Non-anemic			
	Mean ± SD	Median (Min.–Max.)	Mean ± SD	Median (Min.–Max.)		
Age (year)	9.03 ± 6.3	7 (0.5–17)	10.61 ± 4.85	11 (0.5–17)	U = 484.5	0.443
Duration of anemia (month)	10.59 ± 18.5	3 (1–96)	0 ± 0	0 (0–0)	-	-
Hemoglobin (g/dL)	10.38 ± 1.06	10.49 (8.03–12.6)	13.03 ± 1.45	12,73 (10.75–16.45)	U = 49	**<0.001**
Red blood cell count (×10⁶/mm^3^)	4.27 ± 0.53	4.25 (3.03–5.78)	4.69 ± 0.59	4.6 (3.84–6.77)	t = −2.838	**0.006**
Mean cell volume (fL)	76.29 ± 9.89	77.5 (58–99)	83.01 ± 6.44	84 (63–96)	t = −3.220	**0.002**
Folate (ng/mL)	10.3 ± 5.84	8.8 (3.5–23.7)	8.41 ± 3.8	7.21 (4.04–16.94)	U = 333.5	0.342
Vitamin B12 (pg/mL)	408.63 ± 194.64	350.1 (202.5–889)	339.92 ± 228.1	305.2 (23.2–1111)	U = 300	0.091
Iron (µg/dL)	45.93 ± 27.92	42 (8.3–122)	80.18 ± 39.42	87.9 (22–166)	t = −3.629	**0.001**
Iron binding capacity (mcg/dL)	363.06 ± 85.01	373.6 (146.9–530.1)	378.42 ± 30.58	381.6 (332–455.3)	t = −0.902	0.373
Ferritin (ng/mL)	113.9 ± 362.62	22.3 (3.67–2000)	32.34 ± 21.27	26.2 (8.83–91)	U = 353	0.406
FGF21 (pg/mL)	1.24 ± 0.53	1.16 (0.45–3.19)	0.95 ± 0.66	0.73 (0.32–3.47)	U = 337	**0.008**
GDF15 (pg/mL)	2.89 ± 0.73	2.9 (1.53–3.97)	2.9 ± 0.66	3.02 (0.98–3.84)	t = −0.056	0.956
NOS (µg/mL)	1.72 ± 0.59	1.63 (0.61–3.62)	1.43 ± 0.49	1.37 (0.3–2.49)	t = 2.143	**0.036**

Abbreviations: U: Mann–Whitney U Test; t: independent sample *t*-Test; Mean ± SD (standard deviation); Median (min. = minimum, max. = maximum); FGF21: fibroblast growth factor 1; GDF15: growth differentiation factor-15; NOS: nitric oxide synthetase, bold numbers are statistically significant.

**Table 3:** The correlation of FGFR21, GDF15, and NOS parameters with other laboratory values.

		FGF21	GDF15	NOS
Ferritin (ng/mL)	r	−0.069	−0.311	0.019
	*p*	0.611	**0.019**	0.891
Folate (ng/mL)	r	0.191	−0.151	0.313
	*p*	0.159	0.267	**0.019**
Hemoglobin (g/dL)	r	−0.381	0.136	−0.462 *
	*p*	**0.002**	0.287	**<0.001**
Vitamin B12 (pg/mL)	r	0.165	−0.055	0.07
	*p*	0.221	0.682	0.604
Iron (µg/dL)	r	−0.264	−0.095	−0.179
	*p*	0.054	0.493	0.195

Abbreviations: Spearman’s rho Correlation Coefficient; * Pearson Correlation Coefficient; FGF21: fibroblast growth factor 1; GDF15: growth differentiation factor-15; NOS: nitric oxide synthetase, bold numbers are statistically significant.

**Table 4:** Cut points of FGF21, GDF15, and NOS parameters in the prediction of anemia.

	Cut Point	AUC (%95 CI)	*p*	Sensitivity (%)	Specificity (%)	PPV (%)	NPV (%)
FGF21 (pg/mL)	≥0.745	0.690 (0.559–0.822)	0.008	88.24%	53.12%	66.67%	80.95%
GDF15 (pg/mL)	---	0.494 (0.352–0.635)	0.928	---	---	---	---
NOS (µg/mL)	≥1.265	0.647 (0.513–0.781)	0.040	73.53%	56.25%	64.10%	66.67%

Abbreviations: FGF21: fibroblast growth factor 21, GDF15: growth differentiation factor-15, NOS: nitric oxide synthetase, PPV: positive predictive value, NPV: negative predictive value.

## Data Availability

Available for the editorial office on demand.
